# Nano-Enabled Herbal Remedies for Malaria Treatment: A Review of Recent Advances

**DOI:** 10.3390/life16020322

**Published:** 2026-02-12

**Authors:** Chang Xu, Arooj Fatima, Mahreen Fatima, Amjad Islam Aqib, Tean Zaheer, Safia Obaidur Rab, Mohd Saeed, Zeeshan Arif, Kun Li

**Affiliations:** 1 College of Veterinary Medicine, Nanjing Agricultural University, Nanjing 210095, China; 9201710122@stu.njau.edu.cn; 2 Department of Microbiology, Cholistan University of Veterinary and Animal Sciences, Bahawalpur 63100, Pakistan; aroojfatima31202@gmail.com; 3 State Key Laboratory for Animal Disease Control and Prevention, Lanzhou Veterinary Research Institute, College of Veterinary Medicine, Lanzhou University, Chinese Academy of Agricultural Sciences, Lanzhou 730000, China; noormahreen63100@gmail.com; 4 Department of Pharmacology & Toxicology, Cholistan University of Veterinary and Animal Sciences, Bahawalpur 63100, Pakistan; 5 Department of Medicine, Cholistan University of Veterinary and Animal Sciences, Bahawalpur 63100, Pakistan; 6 Department of Entomology, University of Wisconsin, Madison, WI 53706, USA; teanzaheer942@gmail.com; 7 Department of Parasitology, University of Agriculture Faisalabad, Faisalabad 38040, Pakistan; 8 Department of Clinical Laboratory Sciences, College of Applied Medical Sciences, King Khalid University, Abha 61413, Saudi Arabia; srab@kku.edu.sa; 9 Department of Biology, College of Science, University of Hail, Hail 81481, Saudi Arabia; mo.saeed@uoh.edu.sa; 10 Department of Clinical Sciences, Faculty of Veterinary and Animal Sciences, The Islamia University of Bahawalpur, Bahawalpur 63100, Pakistan; zeeshanpgcs@gmail.com

**Keywords:** malaria, *Plasmodium*, mosquito, herbal, ethno-medicine, nanotechnology

## Abstract

Malaria has long been a significant global health concern, listed as a high-priority disease by several global health agencies, despite of several control measures have been put in place. Most widely utilized treatment options for malaria include chloroquine, artemisinin-based combination therapy (ACT), and quinine. However, challenges, such as drug resistance, misdiagnosis, and limited treatment efficacy remain major concerns. Despite ongoing efforts, the development of an effective malaria vaccine is still debatable. Many existing malaria treatments have drawbacks, such as low water solubility, poor bioavailability, and a rise in drug-resistant parasites owing to their non-judicious use, which contributes to increased malaria cases and fatalities. Nanotechnology presents a promising approach to safer and more effective malaria therapy and control. Nanoparticles offer several advantages over conventional treatments, including high drug-loading capacity, targeted delivery, improved biocompatibility, and reduced toxicity in host cells. Green nanotechnology-based antimalarial therapies have demonstrated potential therapeutic benefits, enhanced safety, and cost-effectiveness compared to traditional treatments, ultimately improving patient compliance and treatment outcomes. In this review paper, we discussed non-conventional breakpoints in the malarial life cycle, traditional herbal remedies for malaria, and nanoparticle-based delivery systems. Additionally, we reviewed the antimalarial effects of herbal nano-formulations, their pharmacological and therapeutic potential, drug-resistant malaria, preventive strategies, vector control using green nanomaterials, and the challenges associated with plant-based nanotechnologies. This review suggests nanotechnology-based therapeutics as promising candidates to treat malaria with significant room for applications and commercialization potential in the longer run.

## 1. Introduction

*Plasmodium* species are parasitic protozoa causing malaria, which is common in tropical regions. This major public health disease exhibits a significant rate of illness in addition to the high mortality worldwide, affecting a large number of people. Amidst the constraints with malaria vaccinations, owing to genetic adaptability, inadequate mosquito control, and the non-judicious use of anti-parasitic, the rise in drug-resistant *Plasmodium* has become a huge concern [1]. Anti-malarial medications may be used to prevent and treat malaria, one of the most common and potentially deadly infections in tropical nations, though they may have unfavorable side effects [2]. Infected female *Anopheles* mosquitoes carry *Plasmodium* (apicomplexan parasitic protozoa), which causes one of the most prevalent and deadly vector-borne infectious diseases, including malaria [3]. Genetic variability and the low predictability in the highly adaptable *Plasmodium* contribute to the success of its life cycle, thereby facilitating malarial transmission [4]. Usage of multiple drugs against malaria without careful dosage and regimens could accelerate the rate of mutation of the parasite.

Malaria remains especially challenging to prevent in developing countries owing to the lack of uniform governmental policies, insufficient commitment towards control initiatives, and limited accessibility to effective drugs for malaria prevention and treatment. The disease is on the priority list of the WHO, while reports state that malaria cases with death rates keep increasing drastically over the years. As an example, in 2023 the number of case reports was 263 million, with death rate of 597,000 respectively [5]. In some of the regional areas, death rates were found to be more than 90% in people suffering from malaria, which indicates that contracting the disease could lead to death in certain parts of the world [6]. Chemotherapeutic agents may be used for the treatment and control of malarial infection [7] but are associated with concerns related to the efficacy, safety, and the emergence of drug resistance because of the parasite’s lengthy and intricate life cycle [8]. The use of these drugs is severely restricted because of side effects in some people and the development of drug resistance [9]. Nano-formulations are now being developed to enhance the therapeutic effect of malaria drugs. In recent decades, a variety of nanotechnology-based systems have been studied to enhance the dissolution of physiologically active substances in water. These systems include liposomes, dendrimers, cyclodextrins, magnetic nanoparticles, nano-emulsions, micro-molecules, and rigid lipid nanoparticles, as described in Chaves et al., [10]. This review aims to highlight the antimalarial potential of green alternatives, including herbal remedies and nanomedicines.

## 2. Overview of Plasmodium Life Cycle, Along with Possible Breakpoints for Antimalarial Drugs

The life cycle of *Plasmodium* consists of four stages that involve two hosts: man and mosquito. It has been widely researched and may be divided into the following mechanisms.

**Figure 1:**
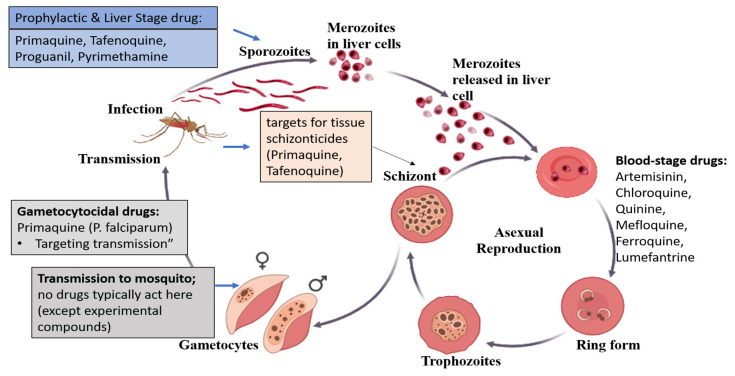
Life cycle of the malarial parasite and possible breakpoints for antimalarial drugs at different stages. Colors, dots, and symbols indicate different *Plasmodium* developmental forms and host cells: small red dots represent parasites (sporozoites, merozoites, and gametocytes), larger circular forms indicate infected host cells, and male (♂) and female (♀) symbols denote sexual stages. Arrows show progression through the parasite life cycle and the sites targeted by antimalarial drugs.

1. Liver stage: The *Plasmodium* parasites are transmitted to the human blood circulation via the female mosquito as sporozoites [11]. After entering the bloodstream, sporozoites migrate to hepatocytes, where they develop into schizonts. The relapse might occur due to the fact that *Plasmodium* species such as *P. ovale* and *P. vivax* remain dormant in the liver as hypnozoites for months or even years after the initial infection. Prophylactic drugs, such as pyrimethamine, proguanil, and others, can be used to target the liver stage of the disease and prevent the development of the malaria parasite. In addition, tissue schizonticides, which are effective against hypnozoites, are needed to make a radical treatment ensuring clearance of the parasite from the liver of the host [12].
2. Blood stage: After around five to ten days, merozoites develop and infiltrate the erythrocytes, causing spiked symptoms such as fever. During their intra-erythrocytic phase, the merozoites transform into trophozoites and schizonts, which are then discharged into the bloodstream to infect un-infected erythrocytes. Chances of severe malarial symptoms and probable mortality could be reduced by antimalarial medications applied at this stage [13,14]. Schizonticides are antimalarials, such as primaquine and pyrimethamine, which are used to target this stage. They work by stopping the illness from spreading to a human host [15]. Combination therapy is an effective way to treat malaria. The World Health Organization authorized the consumption of artemether–lumefantrine, artesunate–mefloquine, artesunate–amodiaquine, and dihydroartemisinic-piperaquine, along with artesunate–sulfadoxine–pyrimethamine as an antimalarial medication mixture for the treatment of *P. falciparum* [16]. Artemisinin derivatives primarily function as therapeutic and transmission-blocking pharmacological interventions in malaria rather than vector-control measures. These compounds rapidly clear asexual blood-stage *Plasmodium* parasites, achieving parasite reduction rates of up to 10,000 parasites/µL within a single 48 h asexual cycle. In addition to their potent blood-stage activity, artemisinin derivatives exhibit activity against immature gametocytes, thereby reducing parasite transmission to mosquitoes. When used in combination therapies, also termed artemisinin-based combination therapies (ACTs), they contribute to the elimination of residual parasites, lowering the risk of recrudescence and delaying the emergence of artemisinin resistance in *Plasmodium* species [17]. Furthermore, their short-term post-treatment prophylactic effect provides temporary protection against reinfection, positioning artemisinin derivatives as a key component of chemotherapeutic and transmission-blocking strategies for malaria control
3. Transmission stage: The gametocytocidal medications utilized to target this stage, such as chloroquine and artemisinin, impede sexual gametocyte formation and growth [14]. Some merozoites undergo differentiation in the bone marrow to form sexual (female and male) gametocytes, which are then consumed by *Plasmodium*-free female *Anopheles* mosquitoes through a blood meal.
4. Mosquito stage: Male and female gametocytes combine in the gut of the female *Anopheles* mosquito to form a zygote, which subsequently matures into an oocyst to eventually form sporozoites that can be transmitted to human hosts. The control of malarial transmission can be achieved by targeting five critical breakpoints: prevention of transmission by the vector; interruption of human–mosquito–human contact; treatment and prevention of infection during the human stage; disruption of parasite development within the mosquito; and modification of environmental and behavioral factors that influence transmission (Figure 1).

## 3. Non-Conventional Breakpoints in the Life Cycle of Malaria

Conventional breakpoints target different stages of the malarial life cycle, whereas non-conventional breakpoints refer to potential intervention points that extend beyond the traditional strategies of vaccination, vector control, and chemotherapy. Such breakpoints emphasize disrupting the transmission, survival, and host interaction at those stages of the life cycle that are usually targets for treatment globally.

Gene-driven systems have the potential to disseminate genetic alterations among mosquito populations, lowering their ability to transmit malaria [18]. Another interesting method is para-transgenesis, which includes the genetic alteration of symbiotic bacteria inside mosquitoes that produce *anti-Plasmodium* effector molecules in order to suppress parasite development [19]. Furthermore, the accidental introduction of *Wolbachia*, a naturally circulating bacterium, into *Anopheles* mosquito populations has been detected to impair their competence to transmit malaria [20]. Exploiting mosquitoes’ dependency on plant sugars for life and reproduction provides another innovative strategy for vector control since it may interfere with their nutrient intake and reproductive success [21,22]. Similarly, host-directed treatments that alter host variables required for parasite survival are an emerging technique in malaria management [23]. Another unique approach can be the interference with exosome-mediated communication. *Plasmodium* parasites employ extracellular vesicles to influence host immune responses and boost their survival [24]. Finally, understanding and mimicking naturally occurring resistance mechanisms, including hemoglobinopathies, may pave the way for the development of novel antimalarial therapies [25].

## 4. Traditional Herbal Remedies for Malaria

Herbal medicines are vital for the discovery of novel pharmaceuticals, and they are already being utilized to treat the effects of malaria as an alternative to conventional antimalarial agents. The development of new medications with better pharmacological and medicinal qualities still heavily relies on traditional medicines. Quinghao, a Chinese herbal remedy, Artemisia annua, and quinine, a species of Cinchona (Rubiaceae), have traditionally been used to treat the symptoms of malaria using traditional medicine [26]. Plants have long been used to treat malaria and reduce the severity of its symptoms, including fever, in many malria affected regions around the world. This is due to the deep-rooted cultural habit and beliefs in traditional medicine, its availability at relatively low cost, its claimed high efficacy, and largely the unknown and underestimated toxicity [27,28]. As an example, one of the medicinal plants in the Celastraceae family, *Maytenus senegalensis,* has been widely studied for its antimalarial potential [29]. Strong antimalarial activity has been demonstrated by scientific investigations into the possible biological activities of *Maytenus senegalensis* entire extract, both in vitro and in vivo [29,30]. Forty-five plant species from twenty-six families and forty-four genera were used to make herbal medicines for malaria and associated symptoms. The most often mentioned plant species were *Senna occidentalis*, *Azadirachta indica*, *Warburgia ugandensis*, *Kedrostis foetidissima*, *Abrus precatorius*, *Vernonia amygdalina*, *Aloe nobilis*, *Chamaecrista nigricans*, and *Mangifera indicat* [31]. The most popular plant element was leaves (67.3%), while the main technique for making herbal remedies was maceration (56%). Traditional medicine is widely accessible, culturally acceptable, and inexpensive, and it is believed to be effective and safe. Since many contemporary allopathic medications, including antimalarial medications, are derived from plants, the examination of plant materials for novel medications against malaria is warranted [32].

Ethnobotanical research has been performed in Ghana on the use of herbal remedies against malaria in various populations and areas. Important botanicals for malaria treatment include *Azadirachta indica* (neem tree) and *Cryptolepis sanguinolenta* (Ghanaian quinine or yellow dye root). Numerous reports on *Cryptolepis sanguinolenta*’s antimalarial qualities exist. Commonly known as the West African antimalarial herb, the plant is locally called Nibima (Twi) [33]. This plant was used only to make Lepiquin, Masada combination, and Nibima herbal medicine. Cryptolepine, the plant’s primary alkaloid, and its analogs, quindoline, biscryptolepine, cryptospirolepine, and cryptoquindoline, which were extracted from the roots of the plant, have been implicated in the plant’s antimalarial properties [34]. In a small open randomized clinical study in adults, a root decoction demonstrated efficacy equivalent to that of chloroquine [35]. More significantly, its main alkaloid, cryptolepine, has demonstrated strong anti-plasmodial action in an in vitro study against *P. falciparum* isolates that are resistant to chloroquine [36]. Therefore, there is a lot of scientific evidence to support the traditional usage of *C. sanguinolenta* to cure malaria. *Azadirachta indica*, commonly referred to as the neem tree (Kingtso-Ga, Liliti-Ewe, and Dua gyanne), is renowned for its many beneficial qualities. Extracts from the leaves, seeds, and stem bark of *A. indica* have demonstrated inhibitory efficacy against *Plasmodium falciparum* in vitro. Additional studies on *A. indica*’s limonoids, gedumin and nimbolide, have also demonstrated efficacy against *P. falciparum* [37], as can be seen in Table 1. However, there was no scientific evidence to support the traditional uses of the following plants: *Ananas sativus*, *Citrus aurantifolia*, *Vitex grandifolia*, *Albizia ferruginea*, *Cola gigantea*, *Teobroma cacao*, *Solanum torvum*, *Aloe schweinfurthii*, *Paullinia pinnata*, *Pycanthus angolesis*, *Adenia cissampeloides*, *Cymbopogon citratus*, *Raphia hookeri*, and *Momordica charantia*. Herbal medicine, on the other hand, integrates several herbs into a single preparation with the goal of providing the body with multidimensional treatment. Therefore, while some of the plants on the list might not directly relate to malaria, their addition will benefit patients’ overall health [38].

## 5. Nanoparticle-Based Delivery Systems in Antimalarial Herbs

**Figure 2:**
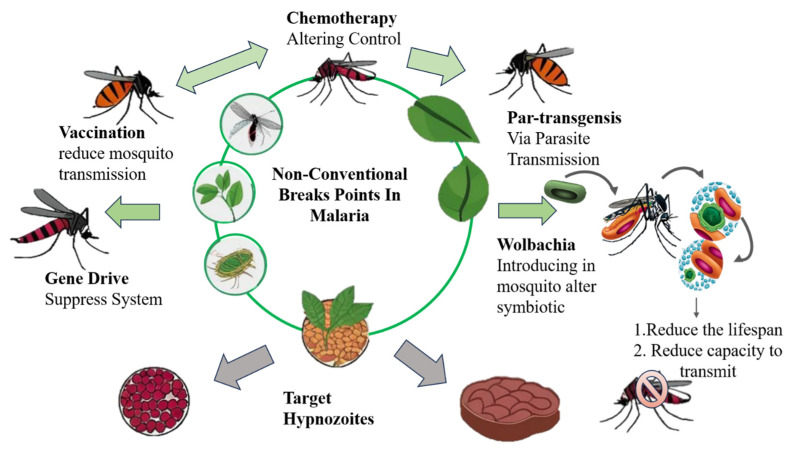
Possible non-conventional breakpoints strategies against the life cycle of mosquitoes. Green arrows indicate the natural transmission or life cycle of the parasite; Gray arrows indicate interventions, blockage, or alternative pathways.

Nanotechnology has a wide variety of uses in pharmaceutical sciences, including precision medicine, drug delivery systems, and diagnostics, because of the distinct structural, physicochemical, and biological characteristics of nanoscale particles [39]. Drugs used in nanoscale medicines are encapsulated or attached to nanostructures that provide several advantages (Figure 2). These advantages include (i) improved oral bioavailability of the drug due to increased aqueous solubility owing to their large surface area yet a nanoscale size, (ii) protection of drugs from gastrointestinal destruction, (iii) controlled drug delivery or targeted delivery, and (iv) improved drug uptake by different cell types [40].

## 6. Antimalarial Effects of Herbal Nano-Formulations

**Figure 3:**
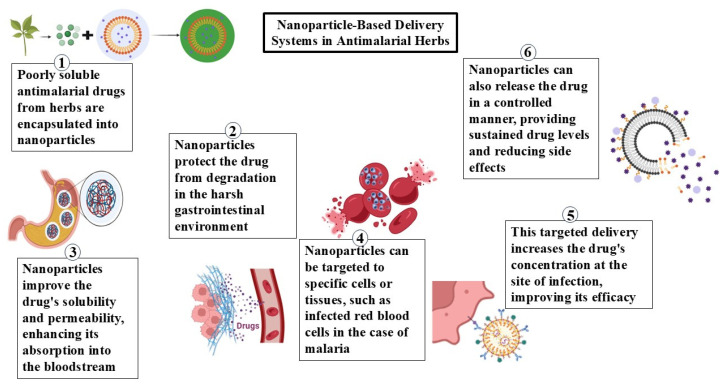
Mechanism of nanoparticles. Overview of nanoparticle-based delivery systems used in antimalarial herbal therapy. (1) Poorly soluble antimalarial compounds derived from medicinal herbs are encapsulated into nanoparticles, where small colored dots represent the drug molecules and the surrounding-colored layers indicate the nanoparticle carrier matrix. (2) Nanoparticles protect these compounds from degradation in the harsh gastrointestinal environment. (3) Encapsulation enhances drug solubility and permeability, improving absorption into the bloodstream, as illustrated by drug-loaded nanoparticles interacting with and crossing biological membranes. (4) Nanoparticles enable targeted delivery to specific cells or tissues, such as infected red blood cells in malaria, with distinct colors differentiating nanoparticles, infected cells, and released drug molecules. (5) Targeting increases drug concentration at the site of infection, thereby improving therapeutic efficacy. (6) Nanoparticles also allow for controlled and sustained drug release, depicted by gradual dispersion of colored drug molecules from the nanoparticle surface, reducing side effects and maintaining stable drug levels.

Herbal remedies have been used for centuries to treat malaria, with both developed and developing nations reporting high usage rates of various herbal medicinal products (HMPs) [41]. Herbal products have been promising sources of new antimalarial agents. The appeal of HMPs and natural medications is ascribed to a number of elements, such as the efficacy claims, whether confirmed or not, and the belief that since they are natural, the derivative treatments might be less toxigenic, safer, and more affordable/available. Additionally, using HMPs is linked to the belief that herbal remedies might be an efficient substitute for conventional medications in situations where conventional treatments do not work [42]. Many HMPs couldn’t outperform conventional anti-malarial drugs in clinical trials despite good therapeutic potential as there are some cons associated such as low aqueous solubility, low intestinal permeability, high molecular size, low oral bioavailability, and inability to cross lipid membranes. Due to the physicochemical constraints of the bioactive ingredients, there are several examples of herbal medicines that exhibit exceptional in vitro activity but extremely low in vivo efficacy [43]. A variety of nanotechnology-based systems have been created for commercially available conventional medications in order to address these issues [44].There are relatively few herbal antimalarial treatments that have been nanoformulated and tested for antimalarial efficacy, including extracts of *Azadirachta indica*, *Momordica charantia*, *Curcuma longa*, and *Artemisia* species. The drug-loaded nano-formulations demonstrated noticeably greater antimalarial effectiveness in vitro and in vivo in each of these investigations. Numerous nano-formulations of antimalarial herbal remedies have been documented, including liposomes, chitosan/lecithin nanoparticles, solid lipid nanoparticles, conventional and polyethylene glycol (PEG) liposomes, nano-suspensions, nano-emulsions, and metal-based nanoparticles [45] (Figure 3).

**Figure 4:**
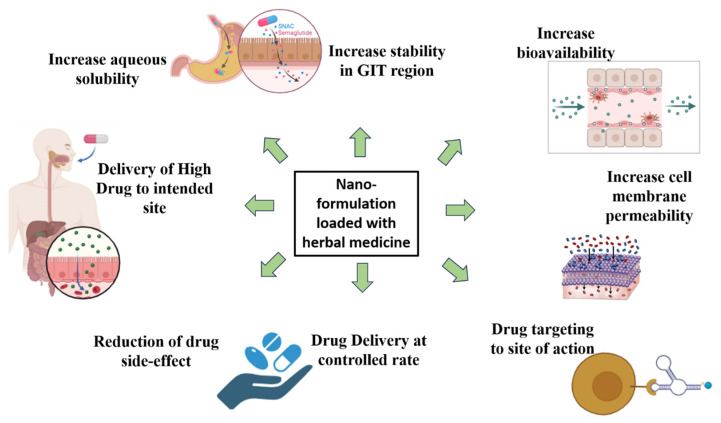
Nanoparticle-mediated enhancement of oral drug delivery and bioavailability. The schematic illustrates the role of nanoparticles in improving drug delivery following oral administration. Colored dots represent drug molecules (free or nanoparticle-encapsulated), while arrows indicate transport and distribution pathways. Nanoparticle encapsulation enhances drug stability in the gastrointestinal (GI) region by protecting active compounds from enzymatic and acidic degradation. Improved interaction with the intestinal epithelium increases cell membrane permeability and facilitates translocation across biological barriers, leading to enhanced absorption. Collectively, these processes result in increased systemic bioavailability, improved therapeutic efficiency, and reduced drug loss during gastrointestinal transit.

The medication is delivered to a precise location by nano-formulations, which also protect it from possible degradation owing to the environment, including enzymes, pH, and the potential for biochemical deterioration [46]. In addition, a relatively low dose of the drug may produce a strong effect due to the ability of the formulation to release the active substance in its active state at the intended site [47]. Many nanosized formulations, including poly lactic-co-glycolic acid (PLGA) nanoparticles, have therefore been created in order to increase the therapeutic effect of curcumin as an antimalarial agent [48] nano-emulsions, hydrogel nanoparticles, solid lipid nanoparticles, nanostructured lipid crystals, chitosan nanoparticles, nano-capsules, lipid-based drug delivery systems, and zwitterionic nanoparticles to address the different challenges of curcumin’s distribution, i.e., site-specific delivery to the impacted tissues, as well as poor absorption and bioavailability. When compared to traditional curcumin administration, the brain’s bioavailability of curcumin was increased by designing nanoparticles (PLGA) that contained curcumin (Figure 4).

Busari et al. [48] developed PLGA nanoparticles loaded with curcumin in different concentrations for antimalarial action. According to the study design, Cur-PLGA demonstrated superior antimalarial activity and safety at low concentrations [48]. PLGA–curcumin adjuvant therapy may therefore be helpful in the treatment of cerebral malaria in humans [49]. Oyeyemi et al. [50] created an encapsulation of curcumin and artesunate that had low toxicity and enhanced anti-plasmodial action. Ghosh et al. [51] extended the lives of *Plasmodium berghei*-infected mice by over two months using a straightforward curcumin nano-emulsion, whereas untreated animals only lived for eight days. Based on the results of this study and the importance of quinine in the treatment of malaria, the nano-capsules developed may be considered as innovative ways to create new formulations containing quinine or curcuma oil or both [52,53,54], as can be seen in Table 2.

## 7. Pharmacological and Therapeutic Potential

### Drug-Resistance

The development of strains of *Plasmodium falciparum* that are resistant to multiple drugs presents serious obstacles to the treatment of malaria. For more than three centuries, quinine was used [60] (Table 3). All malarial species, including strains of *Plasmodium falciparum* that are resistant to chloroquine, are susceptible to the effects of cinchona alkaloids. Mefloquine is a man-made substance similar to quinine, categorized as an aryl-amino alcohol compound. It interacts with free iron in hem, forming harmful complexes that injure the parasite’s membrane. Acute brain syndrome, upset stomach, and prolonged Q-T interval are among the adverse effects. Neuropsychiatric reactions have also been documented [61,62].

Ferroquine is a hybrid of ferrocene–chloroquine antimalarial, which is proving to be more effective in treating chloroquine-resistant *Plasmodium falciparum*. The ferrocene moiety facilitates ferroquine to evade major resistance pathways related to chloroquine, which allows it to have a potent blood-schizonticidal activity. Clinical data reveal better antimalarial activity than chloroquine and an acceptable safety profile, which points to ferroquine as a promising agent in the treatment of drug-resistant malaria. However, pregnant women, people with epilepsy, people on beta-blockers, and people with mental illnesses can face adverse reactions [63]. Nevertheless, some earlier reports exist from the 1990s, indicating low and inconsistent bio-availability of halofantrine. The drug is effective against drug-resistant *Plasmodium falciparum* but presents neuropsychiatric disturbances as notable adverse effect [64].

**Table 3:** Resistance to antimalarial drugs with specific molecular markers.

Gene	Variation	Antimalarial Drug	Risk of Clinical Failure	References
*Pfcrt*	K76T	Chloroquine	Increased risk of treatment failure	[65,66]
*Pfmdr1*	N86Y	Multiple (e.g., Lumefantrine)	Increased risk of treatment failure	[65,66]
*PfATPase6*	Various mutations	Artemisinin	Potentially increased risk	[66]
*Pfk13*	C580Y	Artemisinin	Associated with delayed parasite clearance	[66,67]
*Pfdhfr*	S108N	Pyrimethamine	Moderate risk of treatment failure	[66,67]
*Pfdhps*	G437A, A581G	Sulfadoxine–Pyrimethamine	Moderate to high risk of treatment failure	[66,67]
*cytB*	Various mutations	Atovaquone	Increased risk of treatment failure	[68]

## 8. Green Nanomaterials Against Malaria

Climate change occurring rapidly over the past decades has resulted in ecological disturbances, altered climates that are favorable for vector life cycles, and destruction of natural environments [69]. This scenario has subsequently enabled vector populations to develop at a considerably higher rate and at geographical locations where previously those vector species were not reported. Vector control refers to measures taken against a disease vector to halt its potential to spread associated diseases. This can be achieved by various means, such as limiting the reproductive potential of adult vectors (population size and dynamics and sterile mosquito release), targeting eggs/other terrestrial life cycle stages of the vector, restricting the potential of the vector to reach and attack potential host populations, and enhancing host immunity against vector bites [70]. The possibility of contracting malaria even after vector bites can also be lowered by targeting specific life cycle stages of the *Plasmodium* parasite, as reviewed in the following section.

### 8.1. Green-Metallic Nanomaterials from Plants

Green nanomaterials synthesized by utilizing different parts of the plants have gained currency within the past couple of decades. They are seen as more biogenic, safer, and sustained impact on invertebrate targets with a wider safety profile for mammalian hosts such as humans and animals [71].

**Figure 5:**
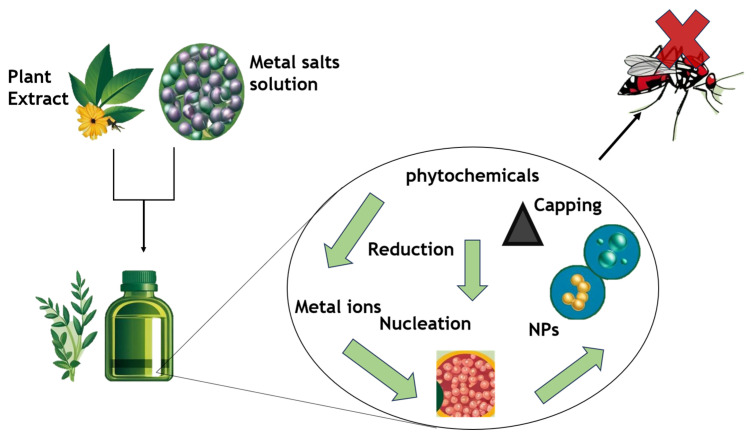
Control of malarial vectors through green synthesis nanoparticles’ metal ions.

Development of a wide range of antimalarial formulations, some metal oxides and metallic nanomaterials—such as silver (Ag), iron (Fe), nickel (Ni), gold (Au), copper (Cu) oxide, and zinc (Zn) oxides and sulfides—have gained popularity due to their unique magnetic properties, elimination of using hazardous chemicals for synthesis, wide surface area, and mild side effects in the hosts. Anti-plasmodial metallic nanoparticles have been produced in an environmentally friendly manner by reducing and stabilizing (capping) the metallic ions using a variety of plants [72,73,74]. Hawadak et al. [75] synthesized AgNPs from the leaves and bark of *Azadirachta indica*, a plant with antimalarial qualities, using aqueous extracts. The anti-plasmodial efficacy of these AgNPs against *Plasmodium falciparum* strains, including the hemocompatibility and stability of the chloroquine-sensitive (CQs)-3D7 and chloroquine-resistant (CQr)-RKL9 strains, was evaluated (Figure 5). The antimalarial activity of the AgNPs made from leaves had an IC50 value of 7.87 μg/mL against the RKL9 strain and 8.10 μg/mL against the 3D7 strain. AgNPs from plant bark and leaves showed improved anti-plasmodial activity when compared to unrefined aqueous extracts of *A. indica*, exhibiting IC50 values of 33.97 μg/mL and 49.64 μg/mL against 3D7; 46.82 μg/mL and 54.10 μg/mL against RKL9.

### 8.2. Plant-Based Oil Nano-Formulations

The use of plant-derived oils in the creation of nano-capsules for the encapsulation of antimalarial medications has grown in popularity as a better substitute for synthetic materials. The pharmacological profiles of these medications have been shown to be considerably improved by this method, providing increased bioavailability, stability, and effectiveness. The researchers in other study, they developed polysorbate-coated Eudragit RS100 nano-capsules containing *Curcuma*-based essential oil as the core for quinine encapsulation. The photostability and efficiency of the drug were enhanced by the nano-encapsulation of quinine. The percentage of photodegradation was considerably lower at 5.1 percent than the 28.8 percent for free quinine. Furthermore, the nano-encapsulated formulation exhibited enhanced therapeutic efficacy, achieving a suppression rate of 8.49% and a mean parasitemia of 61%, compared to 4.88% and 41%, respectively, for free quinine. By favorably affecting the nano-capsules’ zeta potential, this property improves the electrostatic interactions between the anti-parasitic drug and cell membranes [52]. Research has demonstrated the stability and feasibility of Eudragit RS100-based essential oil core nano-capsules since they have a lower density than capric/caprylic triglycerides [76]. Plant oils from seeds such as Brazil nut, olive, sunflower seed, and grape seed, and from plant leaves such as carrot and rosehip oils showed a promising reversible creaming as green nanomaterials. These oils may provide an alternative to standard triglycerides when it comes to forming the plant oil-based core of polymeric nano-capsules [77].

Enhancing the solubility and bioavailability of antimalarial medications derived from plants that are poorly soluble in water has also been the focus of recent developments in antimalarial drug formulation. One potential remedy for these problems is the self-micro-emulsifying drug delivery system (SMEDDS). To improve gastrointestinal motility following oral administration, SMEDDS, which are made of a combination of oil, surfactant (possibly with another co-surfactant), plus the anti-plasmodial activity, can create a micro-emulsion in the gastrointestinal fluid of the host. One of the most successful methods for enhancing the oral absorption and solubility of medications that are poorly soluble in water is SMEDDS [78].

### 8.3. Protein-Based Nanomaterials Against Malaria

The use of albumin in the nano-formulation of antimalarial medications derived from plants, including artemisinin and its derivatives, has also been investigated. Even though combination therapies based on artemisinin have emerged as the mainstay of treatment for malaria while a number of issues remain, including low bioavailability, poor water solubility, and brief half-life, which may limit their potential as anti-protozoal agent. Human serum albumin (HSA) has high potential as an antimalarial drug carrier owing to its ability to target malaria-infected erythrocytes and its excellent bioavailability, biodegradation, lesser toxicity, and being non-immunogenic in humans [79]. Ibrahim et al. created a water-soluble, HSA-bound nanoparticle formulation arising from artemisinin for parenteral administration, tackling the physico-chemical and bio-pharmaceutical issues with the medication showing excellent in vitro antimalarial activity and high entrapment efficiency (97.5%) against the chloroquine-resistant parasites. A promising and more efficient method of treating malaria is the creation of HSA-based nanoparticles, marking a substantial advancement for enhancing selective targeting of malaria within hosts [80].

### 8.4. Nanogels for Drug Delivery of Antimalarials

The nanoparticles based on hydrogels are an effective way to overcome problems with plant-derived antimalarial drugs, like curcumin, through nanogels [11]. In order to improve curcumin absorption and prolong its clearance by possibly circumventing the reticulo-endothelial system, nanogels utilizing a combination of hydroxypropyl methylcellulose with polyvinyl-pyrrolidone were used [81]. The promising formulated plant-based green nanogels’ acute toxicity against *Plasmodium* species and in vivo antimalarial efficacy using mice as a model were evaluated. The parasitemia was significantly (*p* < 0.05) decreased by nanomaterials at a dose of 25 mg/kg body weight when compared to the curcumin control. The nanoparticles were shown to be safe in the acute toxicity testing at a dose of up to 2000 mg/kg body weight. With the potential to help prevent recrudescence and lower the dosage of conventional antimalarial medications, this unique formulation demonstrated promise as an adjuvant system for antimalarial medication [82].

## 9. Challenges Connected with Herb-Based Nanomaterials Against Malaria

In the process of developing plant-based NPs for the treatment of malaria, there are several obstacles and restrictions. In addition to guaranteeing treatment efficacy, safety, and accessibility, these also include obtaining regulatory approval and ensuring consistency and stability in NP preparations. The transition of green nanomaterials from laboratory research data to real-world, clinical applications as treatment of malaria in low- and mid-income areas of the world requires addressing unique obstacles [83].

As *P. vivax* and *P. falciparum* have highest morbidity and mortality rates and contribute to drug resistance, it is imperative to improve the targeted delivery of antimalarials through NPs surface decoration in order to address the challenges posed by the variability in pharmacokinetic profiles, the anti-malarial efficacy, and the safety of these drugs within hosts [84]. So, in order to move NPs from experimental treatments to commercially available therapies, standardizing surface decoration techniques is essential, especially in areas affected by malaria.

These characteristics could work against them because they are not specific to bacteria which increase the likelihood that they will have lethal effects on eukaryotic cells. This is because green nanoparticles have the same size as biomolecules like proteins, DNA, and enzymes, which makes it easier for them to enter living cells and the circulation, from which they can go into other organs. Green particles that are nanoscale in size have the potential to harm cell membranes, oxidatively damage DNA, and disrupt the electron transport chain [85]. The pursuit of antimalarial vaccines employing plant viruses is a challenging endeavor that demands careful advancements to produce safer delivery systems and acceptable stimulants [86]. Standardization and reproducibility pose major challenges to the expansion of plant-based NPs; the variation in phytochemical content and quality demands that the plant materials’ harvesting conditions be standardized in order to guarantee production consistency worldwide. These nanoparticles need to pass via physical, chemical, biological, and microscopic evaluation as well as chromatography and quantitative and qualitative analysis. The issues surrounding unlicensed herbal products draw attention to the serious public health risks that can occur when the herbal ingredients used in them are not properly authorized and authenticated. Adulteration and contamination n of herbal products also pose serious health issues [87].

For testing and verifying the safety of nanomaterials, there are currently no standardized methodologies, which make regulatory approvals more difficult. Furthermore, disparities in the legal systems of various nations may impede cross-border cooperation and the worldwide dissemination of treatments based on nanotechnology (Table 4). So the establishment of standardized regulatory frameworks and standards is essential for the successful clinical and commercial application of plant-based nanoparticles. Especially in areas where malaria is a problem, this would help to ensure that these cutting-edge treatments are safe and available to those in needs while also facilitating more efficient development pathways.

## 10. Malaria and Nanosensors: Advancements and Future Prospects

Globally malaria claimed 409,000 lives and caused over 229 million cases in the year 2019 [90]. Moreover, the major concern is the spread of the disease and its vector mosquitoes beyond endemic areas. Prolong research on malaria and its vectors have helped devise several strategies comprising Integrated Vector Management. It includes emergency preparedness, research, training, continuing education, record keeping, inter-governmental coordination, public education, control activities, disease detection, vector surveillance, program administration, facilities, equipment, financial and economic assessment [91]. Manufacturers may offer limited analytical reviews of Rapid Diagnostic Kits for Malaria (MRDTs), which involve comparing the assays to blood samples infected with malaria parasites. The pathophysiological conditions of total parasite biomass sequestration in the host and the patient’s parasite stage will not be reflected in quantitative data, but qualitative results of antigen activity may be helpful if laboratory-based investigations employ cultured parasites. Additionally, the complications and physical strains that a diagnostic test experience in the field are not replicated in laboratory-based research. Unfortunately, the majority of MRDTs that are being sold commercially, lack peer-reviewed publications that provide independent reviews [92].Comprehensively, the precise and early diagnosis of *Plasmodium* could help prevent serious infections and their deadly outcomes. Additionally, it can help prevent the re-emergence and persistence of infection in areas with low endemicity for malaria. The poor management in malaria-inflicted nations has made quick emergence of antimalarial drug-resistant forms. This emergent situation has rendered the otherwise practical rapid diagnostic test kits (RDTs) to have lower sensitivity and present more false-negative results [93].

**Table 5:** Summary of applications of nano-biosensors for *Plasmodium*.

Target Species/Biomarker	Type of Nanosensors	Features of Nanosensors	Reference
*Plasmodium (P.) falciparum*	ICP-MS-coupled gold nanoparticles	Early ring stage and mixed stage detection LoD = 1.5 pg/mL	[94]
*Plasmodium berghei* *P. falciparum* (Synthetic β-Hematin)	Gold–metal oxide nanoparticles	Separation of interfering signals LoD = 3.3 δ/m	[95]
*P. falciparum* *(Pf-HRP-2)*	Platinum nanoparticles with hydrazine	Non-enzymatic LoD = 2.2 pg/mL	[96]
*P. falciparum*	Magnetic beads and nano-rattles	Differentiates wild-type and resistant strains LoD = 100 attomoles	[97]
*P. falciparum* (pfLDH) *P. vivax* (pvLDH)	Colorimetric	LoD pf = 10.3–12.5 pM LoD pv = 8.3–8.7 pM	[98]
*P. falciparum* (PfHRP-2, PfMSP-1)	Carbon nanofibers on glass microballoons	Simultaneous immune-sensing LoD = 0.025 ng/mL	[99]
*P. falciparum* (PfHSP-70)	Gold nanoparticles	LoD = 2.4 μg/mL	[100]

Symptoms of malaria are overlapped with other febrile conditions. It presents another challenge to the existing clinical kits for diagnosis. The detection of malaria via conventional techniques for *Plasmodium* screening in the field is less effective than nucleic acid-based sensors. Moreover, the staining and microscopic analyses of smears require expertise and time, apart from being labor-intensive. On the other hand, highly sensitive molecular assays, including PCR and ELISA have limited applications amidst field/clinical conditions [100]. In this scenario, nanosensors present solutions to the aforementioned hurdles in the apt diagnosis of malaria, summarized in Table 5. The immobilization of the bio-component is a crucial step in the development of a biosensor. Problems associated with the use of biosensor include thickness and durability of the materials, as well as the chemical and physical parameters (pH, temperature, and pollutants), affecting immobility of the molecules used by the biosensor [101].

## 11. Conclusions

Malaria is an ancient disease whose treatment and prevention in many parts of the globe are still a big challenge. The costs of treatment programs incurred in their elimination could be reduced by formulating herbal remedies in combination with nanomaterials. There is bigger room available in herbal science for control of vector and parasite by adopting nanotechnology. It is need of the hour to rely on the eco-friendly and efficient ways of malaria prevention and control by adopting a holistic approach for both the vector—mosquito—and the highly diverse parasite, *Plasmodium*. There are diverse groups of plants and from them the types of nanoparticles that have shown potential as anti-plasmodium, as proven in various preliminary studies. Preclinical pharmacological studies have also shown the underlying molecular mechanisms of action that can be used to build comprehensive treatment and prevention protocols. However, further research is required to determine optimal dosing regimens and the most effective modes of delivery for the treatment and prevention of different forms of malaria. In addition, potential interactions between patients’ existing medications and herb-based nano-compositions warrant thorough investigation, as this represents a critical and promising area for future research.

## Figures and Tables

**Table 1:** Comparison of some of malaria treatments and herbal remedies.

Type	Uses	Product Names/Plant Species	Dose	WHO Approved	References
Market Products					
Antimalarial	Chloroquine-sensitive malaria	Plaquenil^®^ (hydroxychloroquine)	400 mg weekly prophylaxis	Yes	[30]
Combination Therapy	Uncomplicated malaria	Coartem^®^ (artemether–lumefantrine)	Weight-based dosing (5–40 kg)	Yes	[37]
Artemisinin-based	Severe malaria treatment	Rectal Artesunate (Cipla RAS 100 mg)	100 mg suppository for children < 6 yrs	Yes	[38]
Prophylaxis	Malaria prevention	Malarone^®^ (atovaquone–proguanil)	1 adult tablet daily	Yes	
Chemoprophylaxis	Prevention in endemic areas	Mefloquine	228 mg base weekly	Yes	[38]
Herbal Remedies					
Fever reduction	Symptomatic malaria management	*Vernonia amygdalina* (Bitter leaf)	Leaf decoction (unstandardized)	No	[29]
Antipyretic	Malaria-associated fever	*Azadirachta indica* (Neem)	Leaf extract 2–3× daily	No	[31]
Antimalarial	Traditional treatment	*Aloe nobilis*	Sap or leaf infusion	No	[33]
Symptom relief	Management of malaria symptoms	*Mangifera indica* (Mango bark)	Bark decoction 500 mL/day	No	[34]
Prophylaxis	Community-based prevention	*Warburgia ugandensis* (East African greenheart)	Bark powder mixed with water	No	[35]
Adjunctive therapy	Used with conventional treatments	*Artemisia annua* (Sweet wormwood)	Dried herbal tea 1–2 cups daily	Partially 3	[38]

**Table 2:** Anti-plasmodial activity of plant extract-based nanoparticles.

Plants	Plant Part Extracted	Types of NPs	Size (nm)	Shape	Target *Plasmodium* Strains	Anti-Plasmodial Activity	References
*Azadirachta indica*	Leaves	Silver	4.74–39.32	Spherical	*P. falciparum* (3D7)	EC50: 0.3 μM	[55]
* Ocimum sanctum *	Leaves	Silver	4.74-39.32 nm	Spherical	*P. falciparum* (3D7)	Moderate activity	[55]
*Ocimum sanctum*	Leaves	Silver	4.74-39.32 nm	Spherical	*P. falciparum*	EC50 values ranging from 0.3 μM; enhanced activity when combined with neem extract	[55]
*Sargassum tenerrimum*	Whole plant	Silver	7.71–23.93	Spherical	*P. falciparum*, *P. berghei*	IC50: 7.71 µg/mL (*P. falciparum*), 23.93 µg/mL (*P. berghei*)	[56]
*Cymbopogon citratus*	Leaves	Gold	Not specified	Not specified	Not specified	Control of Anopheles and Aedes larval populations	[57]
* Terminalia bellirica *	leaves	Silver	44.05 nm	cubic	*P. falciparum*	inhibit the parasitized red blood cells (pRBCs)	[58]
* Sargassum wightii *	Not specified	Zinc	Not specified	Not specified	Not specified	LC50 ranged from 4.330–7.430 ppm and 12.278–20.798 ppm	[59]
* Ledebouria revoluta *	Not specified	TiO_2_	Not specified	Not specified	Not specified	larvicidal activity with Lc50(18.960 mg/mL) and LC50 (77.097 mg/mL) against *Aedes aegypti*.	[59]
* Eclipta prostrate *	leaves	Pd-NPs	Not specified	Not specified	*P.* * berghei *	IC50 values achieved were 9.84, 4.49	[59]

**Table 4:** A summary of nanoparticle formulations for the treatment of malaria.

Type of Nanoparticle	Carrier	Application	Therapeutic Outcome	Ref.
Artemisinin-Loaded Nanoparticles	Polymeric, metal-based, lipid nanoparticles	Treatment of malaria	Enhanced solubility, bioavailability, and therapeutic efficacy against *Plasmodium* species	[14]
Lipid Nanoparticles	Glycerophosphorylcholine	Kill parasite in-vivo	Longer retention half-life in the bloodstream, delayed recrudescence and improved survival	[14]
*Curcumin*–Artesunate Nanoparticle	Poly (D, L-lactic-co-glycolic acid) (PLGA)	Treatment of malaria	Significant suppression of *P. berghei* in mice; improved pharmacokinetics compared to free drugs	[50]
Silver Nanoparticles	Silver-based formulations	Mosquito larvicides	Effective against mosquito larvae; significant reduction in the population density of malaria vectors	[59]
Green Synthesized Nanoparticles	Plant extracts (e.g., *Terminalia bellirica*, *Prosopis juliflora*)	Antimalarial activity	Demonstrated anti-plasmodial activity with lower toxicity; eco-friendly alternative	[82]
Plant-Based Nanoparticles	Various plant extracts	Preventative and curative	Effective against *Plasmodium falciparum* at multiple lifecycle stages; enhances drug delivery systems; toxic against mosquito vectors like *An. stephensi*	[82]
Lipid Nanocarriers	Liposomes	Formulation of antimalarial drugs	Enhanced targeting and reduced side effects in drug therapy; improved pharmacokinetic profiles	[82]
Gold Nanoparticles	Gold-coated carriers	Vaccine development	Targeting *P. falciparum* antigen Pfs25; promising results in immunogenicity and protective efficacy	[82]
nanomimics	Not mentioned	block the entrance of the parasite into host red blood cells	Parasite is vulnerable to host‘s defence system and anti-plasmodial therapeutics	[88]
Dendrimers	Not mentioned	Drug delivery	Increase drug loading cpacity and excellent targetting of affected RBCs	[89]

## Data Availability

All data is available in the manuscript.
